# SNP rs1533428 at 2p16.3 as a marker for late-onset primary open-angle glaucoma

**Published:** 2012-06-19

**Authors:** Li Jia Chen, Pancy O.S. Tam, Dexter Y.L. Leung, Alex H. Fan, Mingzhi Zhang, Clement C.Y. Tham, Sylvia W.Y. Chiang, Bao Jian Fan, Ningli Wang, Chi Pui Pang

**Affiliations:** 1 Department of Ophthalmology and Visual Sciences, the Chinese University of Hong Kong, Hong Kong, China; 2 Hong Kong Eye Hospital, Hospital Authority, Hong Kong, China; 3 Prince of Wales Hospital, Hospital Authority, Hong Kong, China; 4 Joint Shantou International Eye Center of Shantou University and the Chinese University of Hong Kong, Shantou, China; 5 Department of Ophthalmology, Harvard Medical School, Massachusetts Eye and Ear Infirmary, Boston, MA; 6 Beijing Tongren Eye Center, Beijing Tongren Hospital, Capital Medical University, Beijing, China

## Abstract

**Purpose:**

To investigate the associations between gene variants in cholesterol 24S-hydroxylase (*CYP46A1*), LIM homeobox transcription factor 1-beta (*LMX1B*), plexin domain containing 2 (*PLXDC2*), toll-like receptor 4 (*TLR4*), transmembrane and tetratricopeptide repeat containing 2 (*TMTC2*), zona pellucida glycoprotein 4 (*ZP4*), chromosome 2p16.3, and primary open-angle glaucoma (POAG).

**Methods:**

We studied 462 POAG patients and 577 controls from three cohorts (Hong Kong, Shantou, and Beijing, China). Twelve single-nucleotide polymorphisms (SNPs) were genotyped in the Hong Kong cohort using TaqMan genotyping assay. Significant associations were validated in the Shantou and Beijing cohorts.

**Results:**

Association of POAG with *TLR4* rs7037117, in a recessive model, was identified in the Hong Kong and Shantou cohorts (both southern Chinese, p_rec_=0.0019) but not the Beijing cohort (northern Chinese). rs1533428 at chromosome 2p16.3 showed a consistent trend of age-specific association in all three cohorts. Genotypes TT + CT conferred a 2.16 fold of significantly increased risk to late-onset POAG (p_dom_=0.00025), but no significant risk to POAG of younger ages of onset in the combined cohort. A joint effect was found between rs7037117 and rs1533428, with carriers of both higher-risk genotypes having a 4.53 fold of increased disease risk (p=0.00028).

**Conclusions:**

Our study reveals discrepant association patterns of 12 candidate SNPs in 7 genes/loci with POAG in Chinese, provides positive replications for POAG markers rs1533428 at 2p16.3 and *TLR4* rs7037117, and suggests that rs1533428 is a putative risk variant for late-onset POAG. The identification of an age-specific association between rs1533428 and late-onset POAG highlights a new genotype-phenotype association in POAG. Further studies are warranted to confirm the age-specific association.

## Introduction

Glaucoma, a heterogenous group of degenerative optic neuropathies [1], is the leading cause of irreversible blindness worldwide [2,3]. Primary open-angle glaucoma (POAG) is a common form of glaucoma. It mainly affects the elderly population, with prevalence increasing with age [4]. Other major risk factors include elevated intraocular pressure (IOP) and genetic variations [5-7].

More than 20 POAG genetic loci have been mapped [6,7] and 3 genes, myocilin [8], optineurin [9], and WD repeat domain 36 [10], identified. Mutations in these genes altogether, however, account for less than 10% of POAG [6,11-13]. In addition, more than 30 POAG-associated genes have been reported [7]. Many of them, however, have failed to be replicated across populations. Recently in a genome-wide association study (GWAS), a single-nucleotide polymorphism (SNP) rs4236601 near the caveolin 1 (*CAV1*) and caveolin 2 (*CAV2*) genes was identified to be strongly associated with POAG in the Icelandic population, and the association was replicated in multiple study populations including Chinese [14]. In another recent GWAS, SNPs at the transmembrane and coiled-coil domains 1 (*TMCO1*) and CDKN2B antisense RNA 1 (*CDKN2B-AS1*) genes were found to be associated with POAG in different Caucasian cohorts [15]. Replication is essential for establishing the credibility of a genotype-phenotype association [16], and it forms an important part of these GWAS [14,15].

Recently, significant associations of variants in the toll-like receptor 4 (*TLR4*) [17], LIM homeobox transcription factor 1-beta (*LMX1B*) [18], and cholesterol 24S-hydroxylase (*CYP46A1*) [19] genes with POAG have been reported in Japanese, British, and French populations, respectively. A combined case-control and linkage study revealed strong association of POAG with 2 SNPs at chromosomal region 2p16.3 in the Afro-Caribbean population of Barbados [20]. In a GWAS report, 6 SNPs at 3 loci were associated with POAG in Japanese [21]. However, lack of association had been reported for the SNPs at chromosome 2p16.3 [22-24], loci detected in the Japanese GWAS [25], and recently the *CYP46A1* [26] and *TLR4* genes [27], whereas associations of *LMX1B* SNPs with POAG have not been replicated in populations other than the initial cohort. In this study, we investigated associations of SNPs in these 7 loci with POAG in study cohorts from three cities of China: Hong Kong, Shantou, and Beijing.

## Methods

### Study participants

This study involved three cohorts of ethnic Han Chinese in different parts of China: a Hong Kong (southern China) cohort recruited from the Hong Kong Eye Hospital and the Prince of Wales Hospital, Hong Kong, and a Shantou (southeast China) cohort from the Joint Shantou International Eye Center, Shantou. The Beijing (northern China) cohort was from the Tongren Eye Center, Beijing [28]. Genetic studies were approved by the institutional review boards of all collaborating institutions and were performed in accordance with the tenets of the Declaration of Helsinki. Written informed consents were obtained from all subjects before the studies.

Complete ocular examinations were performed for each study subject. Diagnosis of POAG were based on: (1) exclusion of secondary causes; (2) Shaffer grade III or IV open iridocorneal angle on gonioscopy; and (3) glaucomatous visual field change detected by Humphrey automated perimeter with the glaucoma hemifield test. The IOP before management, measured by applanation tonometry, was greater than 21 mmHg. The control subjects have no glaucoma or other major eye diseases except mild cataracts and refractive errors. They were recruited from elderly people aged ≥60 years. Participants with known systemic diseases, such as tumor, diabetes, cardiovascular diseases or neurologic diseases, were excluded.

### Candidate SNPs selection and genotyping

Recently reported candidate SNPs around seven genes or loci were selected based on their strong association with POAG: SNP rs7037117 in *TLR4* [17], four risk SNPs (rs944103, rs16929236, rs10733682, and rs867559), a protective SNP rs7854658 in *LMX1B* [18], and rs754203 in *CYP46A1* [19]. SNPs rs1533428 and rs12994401 at the chromosome 2p16.3 region were the most strongly associated with POAG in the Barbados [20], so were SNPs rs693421, rs7081455, and rs7961953 at locus 1q43, 10p12.31, and 12q21.31, respectively, in a Japanese cohort [21].

Genomic DNA was extracted from whole blood using the Qiamp Blood Kit (Qiagen, Hilden, Germany). All of the 12 candidate SNPs were genotyped in the Hong Kong cohort using TaqMan^®^ SNP genotyping assays (Applied Biosystems [ABI], Foster City, CA) on an ABI prism 7000 Sequence Detection System according to the supplier’s instructions. SNPs that showed significant associations were genotyped in the Shantou and Beijing cohorts.

### Statistical analyses

The PLINK program (version 1.07) [29] was used for association analyses unless otherwise stated. Hardy–Weinberg equilibrium (HWE) was assessed for each SNP by χ^2^ test. Allelic, genotypic and model-based (dominant and recessive) associations of the SNPs with POAG were analyzed by χ^2^ test. Because of gender imbalance across our sample sets, we adjusted gender in association analysis using logistic regression. As gender adjustment did not show appreciable effects on the respective associations, only unadjusted odds ratios (ORs) were presented. Gene-wise linkage disequilibrium (LD) between SNPs and haplotype frequencies were tested for disease association using Haploview. For gene-gene interactions, pairwise SNP×SNP interactions were assessed using the epistasis algorithm in PLINK. In parallel, two- and high-order interactions were analyzed using the multifactor dimensionality reduction (MDR) software [30] (version 1.1.0). Data from different cohorts or studies were pooled using a Mantel-Haenszel model with fixed or random effect in the Review Manager software (version 5.0.24; the Cochrane Collaboration, Copenhagen, Denmark). In this study, SNPs giving p-values <0.05 were considered a possible association and included in replication analyses. To conclude the association, we used the Bonferroni method to correct the p-values for multiple testing, using a stringent threshold. First, for correcting p-values in model-based analysis, including allele, full model, dominant and recessive models, a p-value of <0.0010 (0.05÷12÷4) was considered statistically significant. Second, for correcting p-values in stratification analysis, a p-value of <0.00034 (0.05÷12÷4÷3) was considered statistically significant.

## Results

### Characteristics of study subjects

A total of 462 POAG patients and 577 controls were studied: 184 patients and 230 controls from Hong Kong, 102 patients and 147 controls from Shantou, and 176 patients and 200 controls from Beijing (Table 1). For POAG, the age of disease onset may be unreliable because of the absence or unawareness of symptoms at early stages. We resorted, as a surrogate, to use the age at diagnosis (AAD), which was defined as the age at which initial diagnosis of glaucoma was made. All patients in this study have records of no obvious vision impairment at least a year before diagnosis. The ranges of AAD and maximum IOP were large since we did not confine recruitment of study subjects in a narrower range. In contrast, since we included healthy subjects aged ≥60 years as controls, the mean age in the control group of each cohort was significantly greater than in respective patient group (p<0.05, student’s *t*-test). The proportions of gender were significantly different between the case and control groups in the Hong Kong and Shantou cohorts (p<0.05, χ^2^ test), we therefore adjusted gender in association analyses, where applicable, using logistic regression.

**Table 1:** Demographic and clinical characteristics of the study subjects.

** **	** **	** **	**Age at diagnosis years) †**		**Highest recorded IOP (mmHg)**	
**Group**	**Sample size**	Female (%) *	**Range**	**Mean (SD)**	**Range**	**Mean (SD)**
**Hong Kong cohort**						
POAG	184	64 (34.8)	11–88	59.7 (16.6)	22–69	31.0 (9.4)
Control	230	124 (53.9)	60–94	73.5 (7.5)	6–20	13.5 (3.0)
**Shantou cohort**						
POAG	102	20 (20.0)	11–85	45.2 (20.8)	22.5–58	36.5 (9.6)
Control	147	94 (63.9)	63–96	74.0 (6.4)	7–21	12.7 (2.9)
**Beijing cohort**						
POAG	176	38 (21.6)	10–82	38.9 (16.3)	22–70	36.8 (11.0)
Control	200	50 (25.0)	61–85	69.4 (6.0)	10–21	13.0 (3.0)

*There was gender imbalance across our sample sets. We adjusted gender in association analysis using logistic regression and found the adjustment did not affect the associations. †For the controls, age at diagnosis refers to the age at study enrolment. SD: standard deviation; IOP: intraocular pressure.

### Association of individual SNP with POAG

All the 12 SNPs in the Hong Kong cohort conformed to HWE in controls. In allelic association analyses, only *LMX1B* rs944103 showed a mild association (p=0.035; Table 2) which did not withstand Bonferroni correction, corrected p-value (p_corr_)=0.035×12=0.42. *TLR4* rs7037117 was marginally associated with glaucoma in a full model (p=0.049), and it showed a trend toward increased-risk of POAG in a recessive model (p_rec_=0.063, OR_rec_=2.29, 95% CI: 0.94–5.57; after adjustment for gender, p_adj_=0.053). SNP rs1533428 at chromosome 2p16.3 also gave a marginal p-value in the full model (p=0.088). It indicated possible association with POAG in a dominant model (p_dom_=0.030, OR_dom_=1.58, 95% CI: 1.04–2.38; p_adj_=0.015; Table 3). The other 9 SNPs did not show association with POAG.

**Table 2:** Associations of the 12 candidate SNPs with POAG in the Hong Kong cohort.

** **	** **	** **	** **	**MAF (%)**		** **	** **	**Genotype counts †**		** **
**Region**	**Gene**	**dbSNP ID**	Allele *	**POAG**	**Control**	**p-value**	**Odds ratio (95% CI)**	**POAG**	**Control**	**p-value**
1q43	ZP4 ‡	rs693421	T/G	45.9	46.5	0.89	0.98 (0.74–1.29)	37/95/52	48/118/64	0.98
2p16.3	LOC730100 ‡	rs1533428	T/C	44.3	38.4	0.10	1.27 (0.96–1.68)	34/95/55	39/98/92	0.088
2p16.3	LOC730100 ‡	rs12994401	T/C	36.9	34.3	0.46	1.12 (0.84–1.49)	22/91/70	28/101/100	0.49
9q33.1	TLR4	rs7037117	G/A	20.1	20.2	0.99	0.99 (0.71–1.40)	14/46/124	8/77/145	0.049
9q33.3	LMX1B	rs944103	G/A	4.9	2.2	0.035	2.31 (1.06–5.08)	0/18/166	0/10/220	0.029
9q33.3	LMX1B	rs7854658	T/C	0.3	0	0.44	-	0/1/183	0/0/230	0.44
9q33.3	LMX1B	rs16929236	G/A	39.3	40.9	0.67	0.94 (0.71–1.24)	25/94/64	36/116/78	0.85
9q33.3	LMX1B	rs10733682	G/A	26.9	25.3	0.63	1.09 (0.79–1.48)	11/77/96	21/74/134	0.10
9q33.3	LMX1B	rs867559	T/C	30.2	29.8	0.94	1.02 (0.76–1.37)	17/77/90	21/95/114	0.99
10p12.31	PLXDC2 ‡	rs7081455	G/T	15.5	12.8	0.31	1.25 (0.84–1.85)	3/51/130	1/57/172	0.35
12q21.31	TMTC2	rs7961953	A/G	40.8	37.4	0.35	1.15 (0.87–1.53)	30/90/64	29/114/87	0.54
14q32.2	CYP46A1	rs754203	C/T	30.4	33.9	0.29	0.85 (0.63–1.15)	17/78/89	27/100/100	0.56

*Minor allele/major allele; †The genotype counts are presented as homozygote/heterozygote/wildtype; ‡The nearest gene to the SNP; MAF: minor allele frequency; CI: confidence interval.

**Table 3:** Genotype and allele distributions of rs1533428 at chromosome 2p16.3 in different age groups of the study populations

** **	** **	**Hong Kong cohort**		**Shantou cohort**		**Beijing cohort**		** **	** **
Age group*	**Genotype**	**POAG**	**Control**	**POAG**	**Control**	**POAG**	**Control**	**Combined cohort 1†**	**Combined cohort 2†**
Any AAD	TT	34 (18.5)	39 (17.0)	16 (15.7)	26 (17.7)	19 (10.8)	28 (14.0)	** **	** **
** **	CT	95 (51.6)	98 (42.8)	55 (53.9)	60 (40.8)	91 (51.7)	85 (42.5)	** **	** **
** **	CC	55 (29.9)	92 (40.2)	31 (30.4)	61 (41.5)	66 (37.5)	87 (43.5)	** **	** **
p-value (dominant)	** **	0.030	** **	0.074	** **	0.24	** **	0.042	0.0033
OR (95% CI)	** **	1.58 (1.04–2.38)	** **	1.62 (0.95–2.77)	** **	1.28 (0.85–1.94)	** **	1.40 (1.01–1.94)	1.47 (1.14–1.90)
T allele	** **	163 (44.3)	176 (38.4)	87 (42.6)	112 (38.1)	129 (36.6)	141 (35.3)	** **	** **
p-value	** **	0.089	** **	0.31	** **	0.69	** **	** **	** **
OR (95% CI)	** **	1.27 (0.96–1.68)	** **	1.21 (0.84–1.74)	** **	1.06 (0.79–1.43)	** **	** **	** **
AAD < 35 years	TT	0 (0.0)	-	5 (13.9)	-	5 (6.7)	-	** **	** **
** **	CT	10 (58.8)	-	19 (52.8)	-	38 (50.7)	-	** **	** **
** **	CC	7 (41.2)	-	12 (33.3)	-	32 (42.7)	-	** **	** **
p-value (dominant)	** **	0.94	** **	0.37	** **	0.90	** **	0.53	0.59
OR (95% CI)	** **	0.96 (0.35–2.61)	** **	1.42 (0.66–3.05)	** **	1.03 (0.61–1.77)	** **	1.15 (0.74–1.78)	1.12 (0.75–1.67)
T allele	** **	10 (29.4)	-	29 (40.3)	-	48 (32.0)	-	** **	** **
p-value	** **	0.30	** **	0.73	** **	0.48	** **	** **	** **
OR (95% CI)	** **	0.67 (0.31–1.43)	** **	1.10 (0.65–1.86)	** **	0.86 (0.58–1.29)	** **	** **	** **
AAD 35 - 60 years	TT	12 (19.4)	-	4 (10.8)	-	11 (13.8)	-	** **	** **
** **	CT	24 (38.7)	-	22 (59.5)	-	42 (52.5)	-	** **	** **
** **	CC	26 (41.9)	-	11 (29.7)	-	27 (33.8)	-	** **	** **
p-value (dominant)	** **	0.80	** **	0.19	** **	0.13	** **	0.048	0.15
OR (95% CI)	** **	0.93 (0.53–1.64)	** **	1.68 (0.77–3.65)	** **	1.51 (0.88–2.60)	** **	1.56 (1.00–2.44)	1.29 (0.91–1.83)
T allele	** **	48 (38.7)	-	30 (40.5)	-	64 (40.0)	-	** **	** **
p-value	** **	0.95	** **	0.70	** **	0.29	** **	** **	** **
OR (95% CI)	** **	1.01 (0.67–1.52)	** **	1.11 (0.66–1.86)	** **	1.23 (0.84–1.79)	** **	** **	** **
AAD > 60 years	TT	22 (21.0)	-	7 (24.1)	-	3 (14.3)	-	** **	** **
** **	CT	61 (58.1)	-	14 (48.3)	-	11 (52.4)	-	** **	** **
** **	CC	22 (21.0)	-	8 (27.6)	-	7 (33.3)	-	** **	** **
p-value (dominant)	** **	0.00058		0.16	** **	0.37	** **	0.10	0.00025
OR (95% CI)	** **	2.53 (1.48–4.34)		1.86 (0.77–4.48)	** **	1.54 (0.60–3.98)	** **	1.71 (0.90–3.25)	2.16 (1.43–3.26)
T allele	** **	105 (50.0)	-	28 (48.3)	-	17 (40.5)	-	** **	** **
p-value	** **	0.0049		0.15	** **	0.50	** **	** **	** **
OR (95% CI)	** **	1.60 (1.15–2.23)		1.52 (0.86–2.67)	** **	1.25 (0.65–2.39)	** **	** **	** **

*Age groups were defined by age at diagnosis of the patients. The controls were not stratified, and was used as a reference for different age groups of patients. †Data from different study cohorts were combined using Mantel-Haenszel models with fixed effects. Combined cohort 1: combined Shantou and Beijing cohorts; Combined cohort 2: combined Hong Kong, Shantou and Beijing cohorts. AAD: age at diagnosis; OR: odds ratio; CI: confidence interval.

We classified the patients with AAD <35 years as juvenile-onset POAG, between 35 and 60 years as adult-onset POAG, and >60 years as late-onset POAG. Association analyses by comparing each stratum of patients with overall controls showed that genotypes TT + CT of chromosome 2p16.3 rs1533428 conferred a significant increased-risk to late-onset POAG (p_dom_=0.00058, OR_dom_=2.53, 95% CI: 1.48–4.34) but not to juvenile- or adult-onset POAG (Table 3). However, the p-value was at borderline when multiple testing was taken into account (p>0.00034). The other 11 SNPs did not show such a stratified-age association.

**Figure 1:**
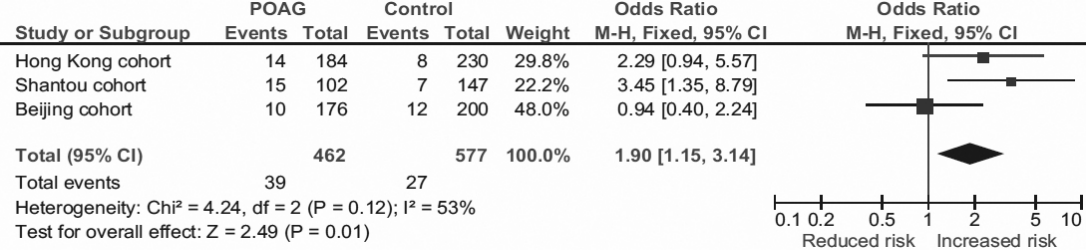
Association of TLR4 rs7037117 with POAG in the recessive model. Squares indicate cohort-specific odds ratio (OR); the size of the box is proportional to the weight of the study cohort; horizontal lines indicate 95% confidence interval (CI); diamond indicates summary OR with its corresponding 95% CI. Meta-analysis indicates a significant association between rs7037117 and POAG.

*TLR4* rs7037117 showed a possible association with POAG in a recessive model in the Shantou cohort. The GG genotype, present in 14.7% of patients and 4.8% controls, conferred a 3.45 fold of increased risk to POAG (95% CI: 1.35–8.79, p_rec_=0.0066, p_adj_=0.0076). In the combined Hong Kong and Shantou southern subjects, the association was enhanced (p_rec_=0.0019, OR_rec_=2.78, 95% CI: 1.46–5.30), although did not reach the significance threshold (p>0.0010). In the Beijing cohort, however, the association between rs7037117 and POAG was insignificant (GG genotype 5.7% in POAG versus 6.0% in controls; p_rec_=0.90; Figure 1).

The higher-risk genotypes of rs1533428, TT + CT, showed a trend toward an increased-risk of POAG in the Shantou cohort (p_dom_=0.074, OR_dom_=1.62, 95% CI: 0.95–2.77; Table 3). The ORs of the risk genotypes also increased with ascending AAD of POAG (Table 3), consistent with the Hong Kong cohort. In the Beijing cohort, the genotype distributions of rs1533428 in patients and controls and the pattern of the stratified-age association were similar to those in the Hong Kong and Shantou cohorts. However, no statistical significance was reached in individual replication cohorts (Table 3). When study subjects were combined in these two cohorts, rs1533428 showed a possible association with POAG (p_dom_=0.042; OR_dom_=1.40, 95% CI: 1.01–1.94). When stratified by AAD, the risk genotypes had an odds ratio of 1.15, 1.56 and 1.71 for early-onset, adult-onset and late-onset POAG, respectively, indicating an ascending trend as observed in the Hong Kong cohort. In the combined subjects from all three cohorts, an enhanced association with POAG was obtained (p_dom_=0.0033, p_corr_=0.036; OR_dom_=1.47, 95% CI: 1.14–1.90). The higher-risk genotypes TT + CT conferred a 2.16 fold of increased-risk toward late-onset POAG (p_dom_=0.00025). The association withstood stringent Bonferroni correction (p<0.00034), yet imposed mild but insignificant risk upon juvenile- (p_dom_=0.59, OR_dom_=1.12) or adult-onset POAG (p_dom_=0.15, OR_dom_=1.29). The TT and CT genotypes individually conferred similar risk to late-onset POAG as each compared to the CC genotype (data not shown), supporting a dominant effect.

### Linkage disequilibrium and haplotype-based association

Linkage disequilibrium between SNPs rs1533428 and rs12994401 at chromosome 2p16.3 was weak in the Hong Kong cohort (D’=0.096 and 0.30 in POAG and controls respectively; r^2^=0.007 and 0.075, respectively). Genotyping rs12994401 in the Shantou cohort revealed weak LD among patients (D’=0.23, r^2^=0.037) and controls (D’=0.25, r^2^=0.053). A common haplotype (C-C) defined by the wildtype alleles conferred a significantly reduced risk of POAG in the Hong Kong cohort (p=0.030, OR=0.74, 95% CI: 0.56–0.97) but not in the Shantou cohort (p=0.32). However, the association in the Hong Kong cohort could not withstand permutation correction (p_perm_=0.07). No haplotype defined by the *LMX1B* SNPs showed a significant association.

### Joint effect of SNPs

Pairwise SNP×SNP interaction analysis in PLINK and two-locus and higher-order interaction analyses in MDR did not reveal statistically significant interactions. Combination of both higher-risk genotypes at rs1533428 and rs7037117 conferred a greater risk to POAG than single risk genotype, indicating an additive effect (Table 4). In particular, in the combined cohort carriers with both higher-risk genotypes had a strong increased-risk of POAG (OR_joint_=4.53; p=0.00028).

**Table 4:** Joint Effect of rs1533428 and rs7037117 on the genetic risk of POAG.

** **	** **	rs1533428** (dominant)**							
** **	** **	**TT + CT (%)**				**CC (%)**			
**SNP**	**Genotype(s)**	**POAG**	**Control**	**p-value**	**Odds ratio (95% CI)**	**POAG**	**Control**	**p-value**	**Odds ratio (95% CI)**
**Hong Kong cohort**									
rs7037117	GG	8 (4.3)	5 (2.2)	0.077	2.91 (0.90–9.37)	6 (3.3)	3 (1.3)	0.080	3.63 (0.87–15.17)
(recessive)	AG + AA	121 (65.8)	132 (57.6)	0.019	1.67 (1.09–2.55)	49 (26.6)	89 (38.9)	1.0	1.0
**Shantou cohort**									
rs7037117	GG	11 (10.8)	3 (2.1)	0.0010	7.61 (1.93–29.53)	4 (3.9)	4 (2.7)	0.44	2.07 (0.48–8.93)
(recessive)	AG + AA	60 (58.8)	83 (56.8)	0.16	1.50 (0.85–2.64)	27 (26.5)	56 (38.4)	1.0	1.0
**Combined cohort**									
rs7037117	GG	19 (6.6)	8 (2.1)	0.00028	4.53 (1.90–10.83)	10 (3.5)	7 (1.9)	0.043	2.73 (1.0–7.45)
(recessive)	AG + AA	181 (63.3)	215 (57.3)	0.0063	1.61 (1.14–2.26)	76 (26.6)	145 (38.7)	1.0	1.0

Joint tow-locus effect between SNPs rs1533428 and rs7037117 for POAG in southern Chinese. Only one model was used. The joint odds ratios were estimated for the combination of genotypes with at least one at-risk genotype of the SNP rs1533428 (in dominant model) or rs7037117 (in recessive model) compared with the combination of both non-risk genotypes.

## Discussion

In this investigation of 12 candidate SNPs, association with POAG was found for *TLR4* rs7037117 (P_rec_=0.0019), which was initially identified in a Japanese population [17], in the Hong Kong and Shantou cohorts of southern Chinese, but not in the Beijing cohort of northern Chinese. In a recent study, no association was found between rs7037117 and normal tension glaucoma in a Korean population [27]. These findings suggest a geographic diversity in the association. Moreover, while rs7037117 was associated with normal tension glaucoma in Japanese [17], our results suggest that *TLR4* may also be implicated in high-tension POAG. Such geographic and phenotypic diversities might have resulted from other yet-to-identify genetic and/or environmental factors. SNP rs1533428 at chromosome 2p16.3 showed similar trends of association with POAG in all our three Chinese cohorts, although a significant p-value (p<0.05) was only detected in the Hong Kong cohort. Moreover, combined analysis of the discovery and replication cohorts, a common practice in genetic association studies [14,15], had led to a more significant P-value, indicating a positive replication. Furthermore, rs1533428 and rs7037117 together had an additive effect, supporting the polygenic property of POAG. Except for these two, we did not find significant association between POAG and other candidate SNPs at or around the *CYP46A1*, *LMX1B*, *PLXDC2*, *TMTC2*, and *ZP4* genes. Our findings exemplify the ethnic diversities in POAG genetics and the importance of replication analysis for new candidate genes of POAG.

**Figure 2:**
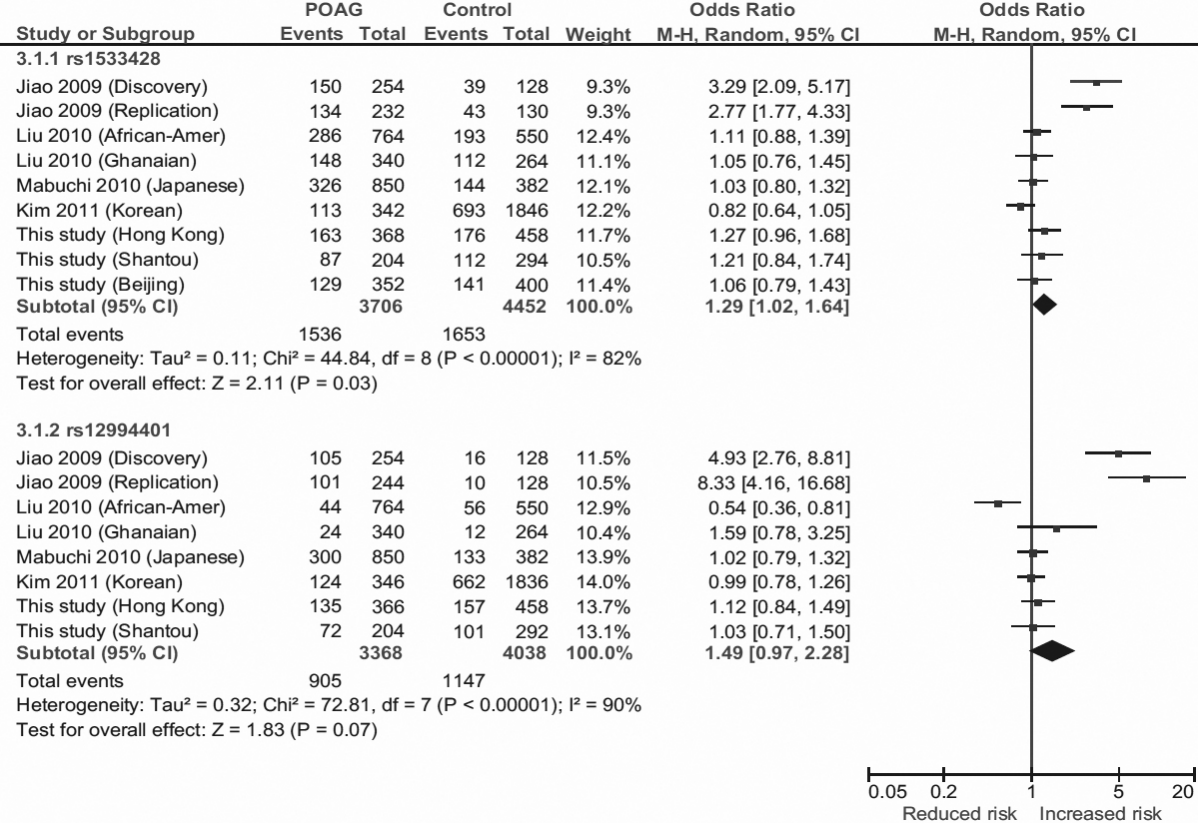
Allelic association of SNPs rs1533428 [T] and rs12994401 [T] at 2p16.3 with POAG. Squares indicate cohort-specific odds ratio (OR); the size of the box is proportional to the weight of the study population; horizontal lines indicate 95% confidence interval (CI); diamond indicates summary OR with its corresponding 95% CI. Meta-analysis indicates significant associations of rs1533428 (p=0.03), but not rs12994401 (p=0.07), with POAG.

Ethnic heterogeneity exists in the associations of rs1533428 and rs12994401 at chromosome 2p16.3 with POAG (between-study variance Tau-square=0.10, p<0.00001; Figure 2). In the Afro-Caribbean population of Barbados, these two SNPs are strongly associated with POAG, with rs12994401 [T] (OR_hom_>33.0) conferring a stronger risk than rs1533428 [T] (OR_hom_>5.5). They are in strong LD (D’=0.72) [20]. In contrast, in the African-American and Ghanaian populations rs12994401 [T] conferred a reduced-risk of POAG (OR=0.53, p=0.003) in the African-American population but a trend of increased-risk (OR=1.6, p=0.22) in the Ghanaian population. However, rs1533428 [T] conferred a mild but nonsignificant risk to POAG (OR≥1.05, p>0.10) in both populations. These two SNPs were in weak LD in either population [22]. In contrast to the African populations, we found that rs12994401 [T] was not significantly associated with POAG in Chinese, whereas rs1533428 [T], mainly in a dominant model, conferred a significantly increased risk, and there is low LD between them. In a Japanese population, rs12994401 [T] and rs1533428 [T] have a very mild trend toward an increased risk of POAG (OR≥1.02), although no significance was reached. Notably, after adjusting for age, gender, IOP and refractive error, rs1533428 [T] remained a trend of increased-POAG risk (OR=1.05), whereas rs12994401 [T] showed a reverse tendency (OR=0.93) [23]. Recently in a Korean cohort, both the rs1533428 [T] and rs12994401 [T] showed a lack of association, and the odds ratios were to a reversed direction [24]. In view of such discrepancies, we conducted a meta-analysis of all reported studies on these two SNPs, involving over 1,600 POAG and 2,000 controls (Figure 2). rs1533428 [T] showed a same trend of increased POAG-risk across all study populations except Korean, giving a combined p-value of 0.035. In contrast, rs12994401 showed an insignificant association (P_meta_=0.067). These findings together suggest that rs1533428 is more likely to be a risk marker for POAG at chromosome 2p16.3, but with high degree of ethnic diversities.

Linkage of the chromosome 2p locus in 5 families with autosomal dominant POAG had been reported [20]. Also, genetic linkage between the chromosome 2p15–16 region with autosomal dominant POAG has been identified in six white and one Afro-Caribbean families [31], and in two Chinese families [32]. Thus, the chromosome 2p16 region may harbor one or more disease genes for POAG. However, no functional gene at chromosome 2p16.3 surrounding rs1533428 has been annotated except for a hypothetical gene *LOC730100*, which encodes a predicted protein with some homology to the TRF1-interacting ankyrin-related ADP-ribose polymerase. The latter plays a regulatory role in telomere length [20,33], which is associated with cellular senescence and apoptosis [34].

We have identified a stratified-age association between POAG and rs1533428, where the TT and CT genotypes were significantly associated with late-onset POAG but not adult- or juvenile-onset POAG. We obtained consistent findings across three cohorts from different geographical locations in China, indicating no artifact of a cohort effect. Also, as all our study subjects have no known systemic diseases, such age-specific pattern was unlikely attributed to interactions with other age-related morbid conditions. Thus, rs1533428 is likely a susceptibility genetic marker for late-onset POAG in Chinese. The higher-risk genotypes conferred a 2.16 fold of increased risk to late-onset POAG in a dominant model, similar to the dominant OR in the study of Jiao et al. [20] (OR_dom_=2.48 in the combined group), whose patients were mainly late onset (mean age >70 years) [20]. It is therefore evident that rs1533428 correlates with late-onset POAG. Interestingly, however, in a Korean study population, the SNP was not associated with POAG in patients aged above 62.5 years [24]. This further highlights the ethnic diversities in this association and the possibility of other modifying genetic and environmental risk factors.

Similar age-specific genetic correlation had been found in glaucoma and other diseases. For example, myocilin glaucoma is frequently diagnosed between 20 and 40 years of age [35]. Allingham et al. [36] established a linkage to chromosome 15q11–13 in families with early-onset POAG. SNP rs4293393 in the uromodulin (*UMOD*) gene was strongly associated with chronic kidney disease in patients aged above 70 [37]. deleted in malignant brain tumors 1 (*DMBT1*) SNPs rs2981745 and rs11523871 significantly increased breast cancer risk only in women aged above 60 years [38]. The biologic relevance of the stratified-age association found in our POAG patients is not known. Late-onset POAG is more likely to follow the common disease common variant mechanism, whereas juvenile-onset POAG may be caused by gene mutations with high penetrance [8]. Hence, it is possible that certain common polymorphisms, e.g., rs1533428, act predominantly on late-onset POAG. Since penetrance of susceptibility polymorphisms are usually low, other risk factors are needed to determine the development of disease, such as an older age [1,5]. Therefore the age-specific association may suggest an age-dependent penetrance of rs1533428 in the development of POAG.

This study is a direct association investigation between patients and controls. However, we included only a limited number of candidate SNPs from each gene/locus. A more exhaustive investigation, e.g., haplotype-tagging SNP analysis, could provide additional useful genetic information. Also, the sample size in each stratum was not large, thus limiting the statistical power to detect significance in individual cohorts. Furthermore, this study is a replication and the stratification analysis by age was retrospective, which is suboptimal for testing new associations. Therefore further prospective discovery studies specifically aiming at testing the age-specific association between rs1533428 and POAG are warranted.

In summary, we have identified discrepant association patterns of 12 candidate SNPs in 7 genes/loci with POAG in the Chinese population, exemplifying ethnic diversities of those associations and the need of replications in genetic studies. We have, for the first time, provided positive replications for SNPs rs1533428 at chromosome 2p16.3 and *TLR4* rs7037117 in Chinese and detected an additive effect between them. The identification of an age-specific association between rs1533428 and late-onset POAG may highlight a new genotype-phenotype association in POAG.

## 
